# Eye damage due to cosmetic ultrasound treatment: a case report

**DOI:** 10.1186/s12886-018-0891-2

**Published:** 2018-08-29

**Authors:** Yuanyuan Chen, Zhongyu Shi, Yin Shen

**Affiliations:** Eye Center, Wuhan University Renmin Hospital, 238 Jie Fang Road, Wu Chang District, Wuhan, 430060 China

**Keywords:** Rejuvenation, Acute increase of IOP, Myopia, Accommodation spasm

## Abstract

**Background:**

Rejuvenation of aging eyelids is one of cosmetic changes to the individual to create the appearance of youth. Tightening treatment of eyelid by ultrasonic heat could possibly develop acute eye injury, including acute increase of IOP, cataract and rarely myopia.

**Case presentation:**

A case report of rejuvenation tightening treatment caused eye injury with 6 months’ follow-up. All examinations were performed at a university teaching hospital. A healthy 32-year-old Asian woman had pain, photophobia and blurred vision in the right eye after rejuvenation tightening eye brow treatment. Intraocular pressure (IOP) was 31 mmHg in the right eye. Tyndall phenomena were observed. Visual acuity of the right eye dropped to 20/200 (from 20/20), with best-corrected visual acuities (BCVAs) 20/20. An iris pigment detachment was found. Neuro-ophthalmic examination was relative afferent pupillary defect (RAPD) positive with pericentral scotoma in the right eye, indicating optic nerve damage. In the optical quality analysis system (OQAS) exam, the objective scatter index (OSI) was 1.0 in the right eye and 0.7 in the left. Clearing additional plus lens power was difficult for this patient, indicating accommodation spasm in the right eye.

**Conclusions:**

Rejuvenation with intense-focused ultrasound (IFUS) could cause heat injury, leads to acute increase of IOP. Heat damage in zonular fibers could cause accommodation spasm and myopia. Eye injuries caused by IFUS has been seldom reported. We recommend that cosmetic treatment in the eye area should be highly aware of side effect.

**Electronic supplementary material:**

The online version of this article (10.1186/s12886-018-0891-2) contains supplementary material, which is available to authorized users.

## Background

Cosmetic ultrasound treatment is a large industry, offering superficial treatments to improve a person’s appearance. Rejuvenation of aging eyelids is one of cosmetic changes to the individual to create the appearance of youth. We herein reported an Asian woman who developed acute increase of IOP and acquired myopia after she received rejuvenation tightening treatment through intense focused ultrasound (IFUS) treatment. IFUS is an energy modality that can be focused and penetrates deeper in the tissue to cause selective thermal coagulation within the focal region of the beam [1]. The generated heat causes tightening effect of the skin, but can also lead to eye injury, if applied improperly.

## Case presentation

A healthy 32-year-old Asian woman presented to the emergency department with pain, photophobia and blurred vision in the right eye. There were no associated illnesses, history of retinotoxic exposures (medications, light), or family history of eye disorders. Written informed consent was obtained; the procedures adhered to the Declaration of Helsinki, and the study was approved by the institutional review board of the Wuhan University Renmin Hospital.

**Fig. 1:**
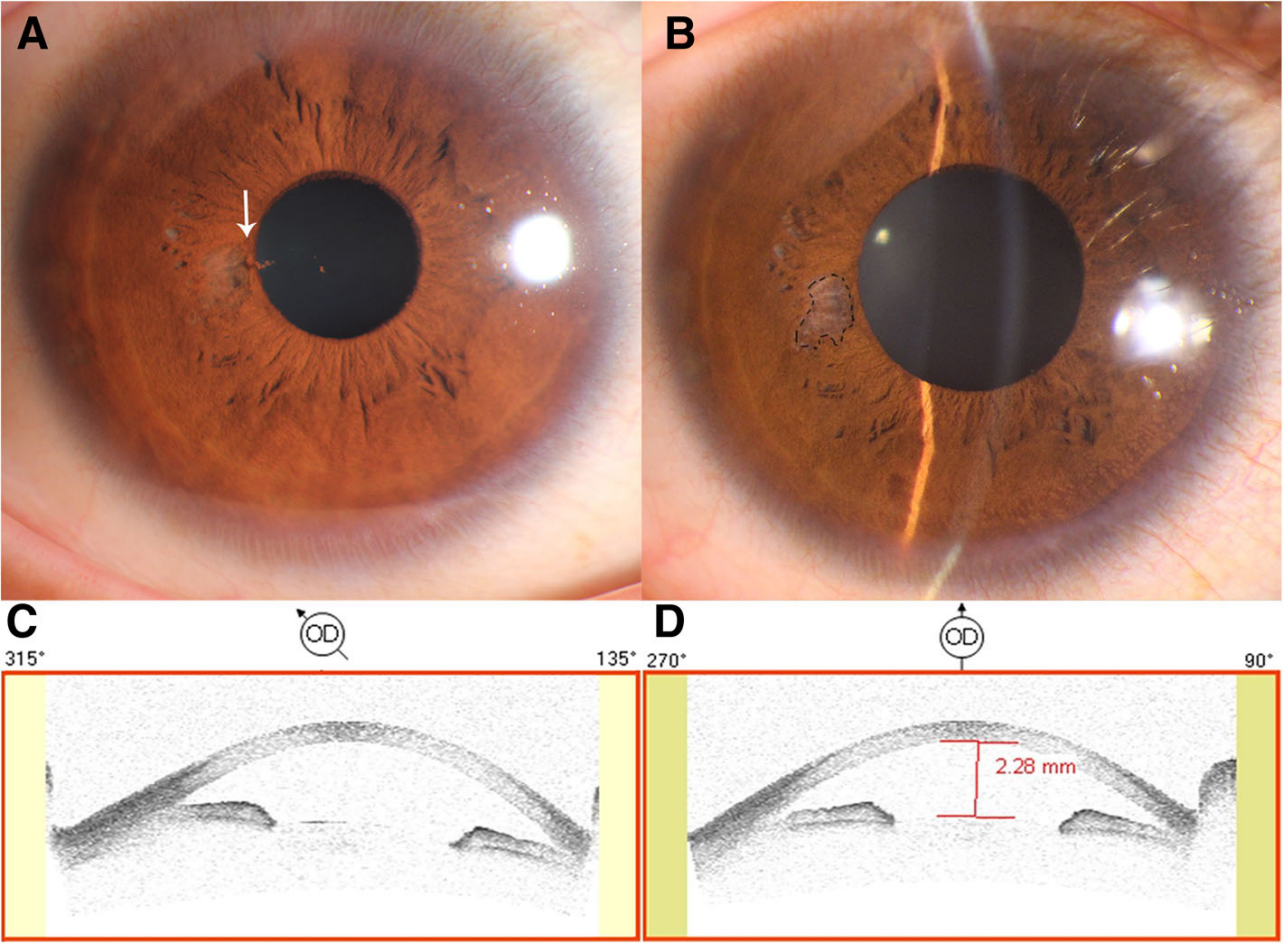
IFUS induced iris pigment detachment. (**a**) anterior eye photo. White arrow: detached iris pigment floating in the anterior chamber. **b** One-week follow-up. Black circle: heat damaged iris; (**c**, **d**) Anterior segment optical coherence tomography (ASOCT) showed shallow anterior chambers at superior lateral in the right eye

Her uncorrected visual acuity was 20/200 in the right eye, 20/25 in the left eye. Intraocular pressure (IOP) was 31 mmHg in the right eye and 16 mmHg in the left. Neuro-ophthalmic examination was RAPD positive in the right eye. Mid-dilated fixed pupil in the right eye (Φ ≈ 4 mm). Tyndall phenomena were observed, vitreous were normal without cells; fundus examination results were also normal. The anterior segment image present with iris pigment detachment at 9 o’clock in the right eye (Fig. 1a-b). Results of anterior segment optical coherence tomography (ASOCT) showed slightly shallow anterior chamber in right eyes (Fig. 1c-d). The unharmed left eye also show slightly shallow anterior chamber (Additional file 1: Figure S1). On subsequent questioning, the patient disclosed that she received an intense-focused ultrasound (IFUS) in a cosmetic surgery center to lift and tighten the upper eyelid. Ultrasonic probe was applied at the eyebrow area. She immediately complained of painful blurry vision, the treatment was stopped and she was transferred to hospital.

**Fig. 2:**
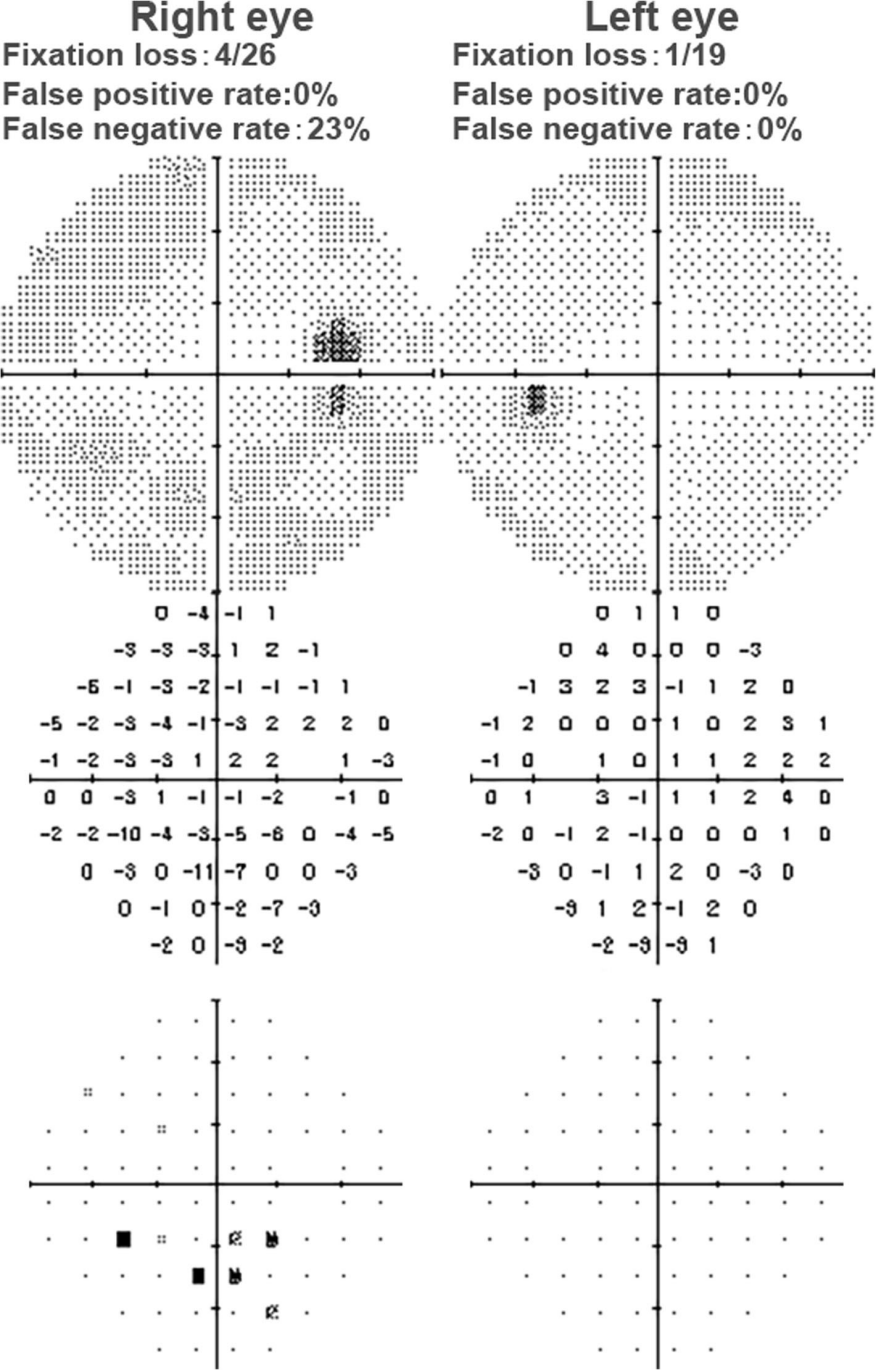
Pericentral scotoma in the right eye indicating optic nerve damage caused by acute increase of IOP after the IFUS treatment

After anti-glaucoma treatment for 1 day, IOP of the right eye dropped to normal range (21 mmHg). Uncorrected visual acuity in the right eye was improved to 20/160. Best-corrected visual acuity (BCVA) of right eye was 20/20 with refraction of − 1.50 DS/− 1.0 DC × 165. At 3 days follow-up, spectral-domain optical coherence tomography (SD-OCT) was performed using an SD-OCT/scanning laser ophthalmoscope system (Heidelberg Engineering). Automated light-adapted static perimetry (HFA II-I; Carl Zeiss Meditec) using a 30–2 protocol confirmed a pericentral scotoma corresponding to the abnormality in the right eye (Fig. 2), which could be causal by acute increase of IOP induced optic nerve edema; the left eye finding was unremarkable. Standard full-field flash electroretinography (ERG), and visual evoked potential (VEP) was normal, ruling out retina and brain disease. Optical Quality Analysis System (OQAS) exam showed that the objective scatter index (OSI) was 1.0 in the right eye and 0.7 in the left, modulation transfer function (MTF) cut-off was 23.831 in the right eye and 28.694 in the left; indicating comparable worse vision quality in the right eye (Additional file 2: Table S1), at 1 month’s follow-up.

At presentation to us on the 3 months of her symptoms, the patient still complained with headache, blur vision with photophobia in the right eye. Her iris damage was remained, with IOP measurement in normal range. Acquired myopia became her major complain. A comprehensive eye exam was further performed by an optometrist. Negative and positive relative accommodation (NRA/PRA), and accommodation amplitude was measured. Clearing additional plus lens power was difficult for this patient, indicating accommodation spasm in the right eye.

Accommodative spasm is a rare condition occurring in children, adolescents, and young adults. It can be caused by trauma [2], emotional problems, and other causes. In our case, further exam confirmed her accommodative spasm which was partially reversed by cycloplegia drops and bifocals.The patient was put on 1% tropicamide eye drops once a day and flipper lenses training was applied to treat her accommodation spasm. After 1 month, the patient achieved better uncorrected vision acuity at distance in the right eye (20/40) (Additional file 3: Table S2).

**Fig. 3:**
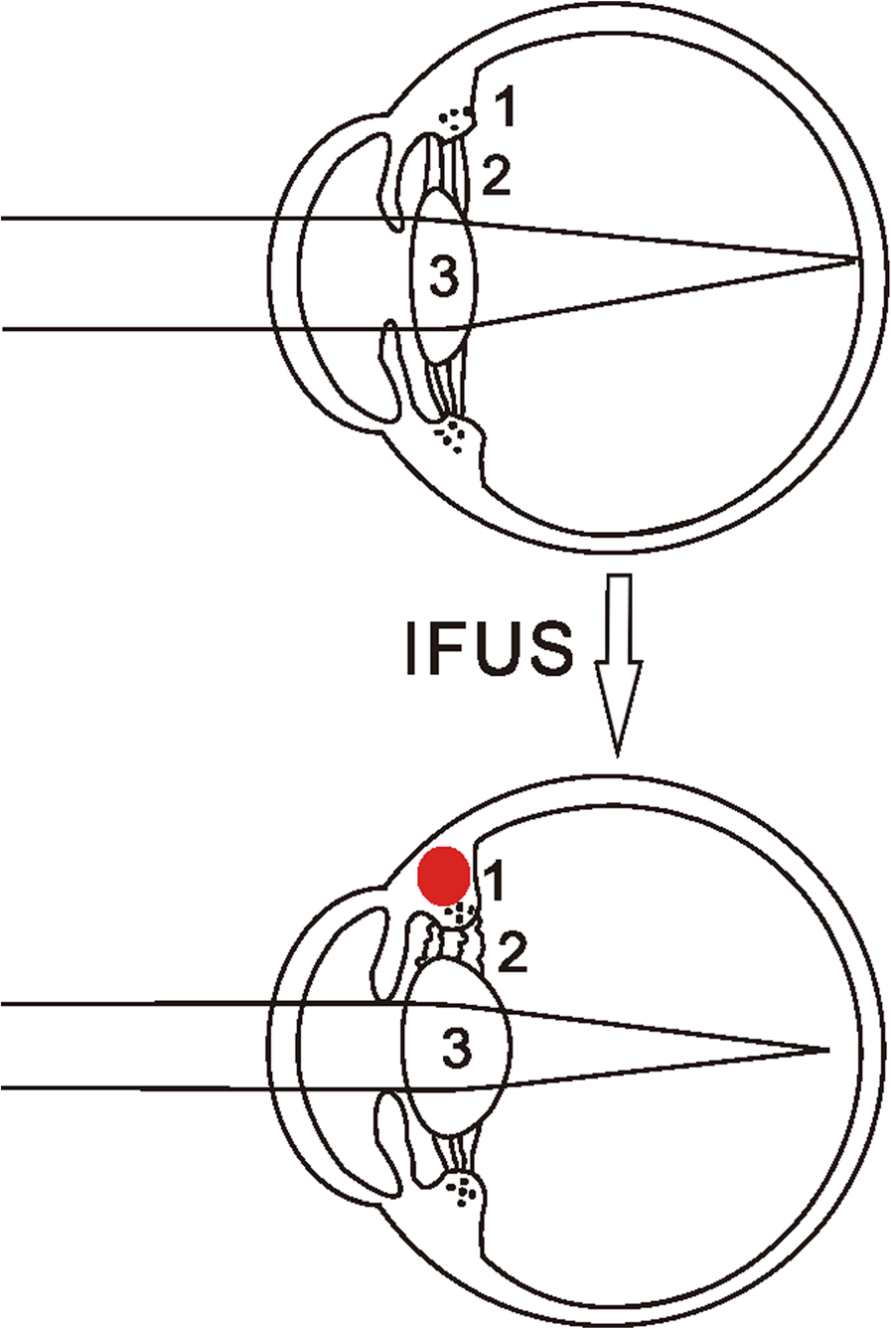
IFUS induced acquired myopia. The acoustic energy rays might cause dysfunction of the ciliary muscle, which affected the zonular tension, induced acquired myopia. The excessive curvature of refractive lens surface leads to curvature myopia and accommodation spasm. 1, ciliary muscle. 2, zonular fiber. 3, the lens

Safety and efficacy of IFUS in the aging eyelids have been studied and reported in the previous study [3]. Tightening of infraorbital laxity and skin can be achieved using the IFUS, is performed by heating the dermis and underlying tissue, where protein around the focal point will reach over 65 °C and denatured within milliseconds [4]. After the initial heat effects, the skin initiates a wound healing response, resulting in the formation of new collagen, which provides longer-term tightening of the skin [5]. In this case, the acoustic energy rays might have threaded through the eyelid, caused dysfunction of the ciliary muscle, which affected the zonular tension, causing acute increase of IOP and acquired myopia. The excessive curvature of refractive lens surface leaded to curvature myopia and accommodation spam (Fig. 3). Heat-caused injuries may be associated with a transient IOP increase due to acute trabecular meshwork changes.

## Discussion and conclusion

Safety and effectiveness of IFUS have been studied on the neck and face, including infraorbital area [6, 7]. Ultherapy (Ulthera Inc., Mesa, AZ, USA) has received FDA clearance for eyebrow lift [8]. To the best of our knowledge, Eye damage caused by IFUS has been seldom reported [9]. Side effects such as skin pain, transient erythema, and purpura have been reported in the previous study [6]. Whereas heat produced by the devices could possibly cause severe damage, to the tender eye tissue, including iris damage, zonal damage, cataract, acute increase of IOP, even possibly retina damage or optic nerve damage.

Here we report a case that caused acute transient increase of IOP and myopia. The patient myopia seems to be very different than the others. Accommodation spasm is a rare condition characterized by a sudden increase in myopia. In our case spasm of accommodation (also known as a ciliary spasm) is a condition in which the ciliary muscle of the eye remains in a constant state of contraction that could be caused by IFUS tightening. In a state of contraction, the ciliary muscle cannot relax when viewing distant objects (Fig. 3). Whereas rapidly acting parasympatholytic drug is useful in producing cycloplegia of short duration. After cycloplegia, relaxed ciliary muscle allowed the lens zonules and suspensory ligaments to pull on the lens, flattening it, and then our patient had partially recoverd vision from a distance.

Rejuvenation of aging eyelids is one of cosmetic changes to the individual to create the appearance of youth, also could possibly induce severe eye disease, including acute increase of IOP, acquired myopia, even possibly cataract, which requires a large amount of attention. Ultrasonic probe applied around the eyebrow should be strictly forbidden to avoid eye damage.

## Additional files

Additional file 1: **Figure S1.** Anterior segment optical coherence tomography (ASOCT) showed shallow anterior chambers at superior in the left eye. (TIF 2916 kb)

Additional file 2: **Table S1.** Optical Quality Analysis System (OQAS) at one-month follow-up, indicating comparable worse vision quality in the right eye. (DOCX 92 kb)

Additional file 3: **Table S2.** Intraocular pressure (IOP) and uncorrected visual acuity (VA) and best-corrected visual acuities (BCVAs) of this patient. OD: right eye. OS: left eye. (DOCX 30 kb)

## Abbreviations

ASOCT: Anterior segment optical coherence tomography
BCVAs: Best-corrected visual acuities
ERG: Electroretinography
IFUS: Intense-focused ultrasound
IOP: Intraocular pressure
MTF: Modulation transfer function
NRA/PRA: Negative and positive relative accommodation
OQAS: Optical quality analysis system exam
OSI: Objective scatter index
RAPD: Relative afferent pupillary defect
SD-OCT: Spectral-domain optical coherence tomography
VA: Uncorrected visual acuity
VEP: Visual evoked potential
