# Supplementary figures and images for: Eye damage due to cosmetic ultrasound treatment: a case report
Source: BMC Ophthalmol. 2018 Aug 29;18:214. doi: 10.1186/s12886-018-0891-2 (PMC6114535; doi:10.1186/s12886-018-0891-2)

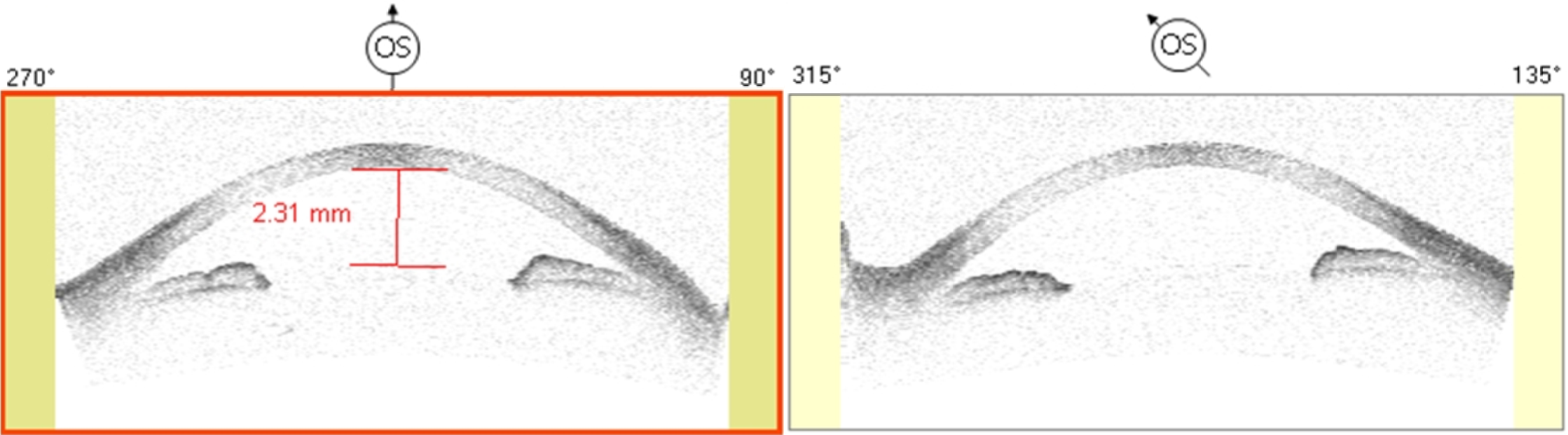

Supplement: Supplementary file 1 — Figure S1. Anterior segment optical coherence tomography (ASOCT) showed shallow anterior chambers at superior in the left eye. (TIF 2916 kb) [file 12886_2018_891_MOESM1_ESM.tif]
