# Supplementary material for: Eye damage due to cosmetic ultrasound treatment: a case report
Source: BMC Ophthalmol. 2018 Aug 29;18:214. doi: 10.1186/s12886-018-0891-2 (PMC6114535; doi:10.1186/s12886-018-0891-2)
Supplement: Supplementary file 2 — Table S1. Optical Quality Analysis System (OQAS) at one-month follow-up, indicating comparable worse vision quality in the right eye. (DOCX 92 kb) [file 12886_2018_891_MOESM2_ESM.docx]

|  | Right eye | Left eye |
| --- | --- | --- |
| OSI | 1.0 | 0.7 |
| MTF cut-off (c/deg) | 23.831 | 28.693 |
| Double-pass image | **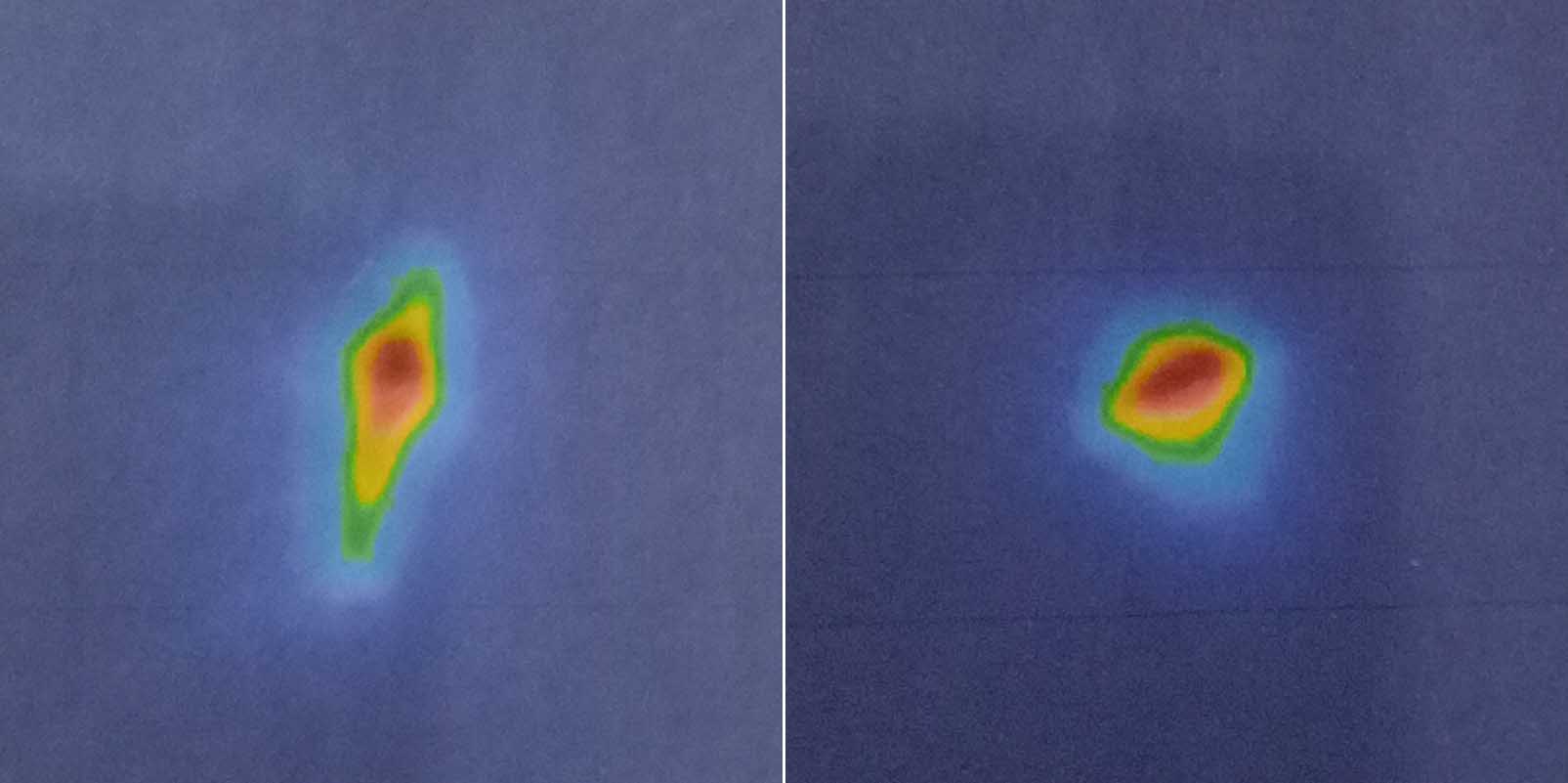** | **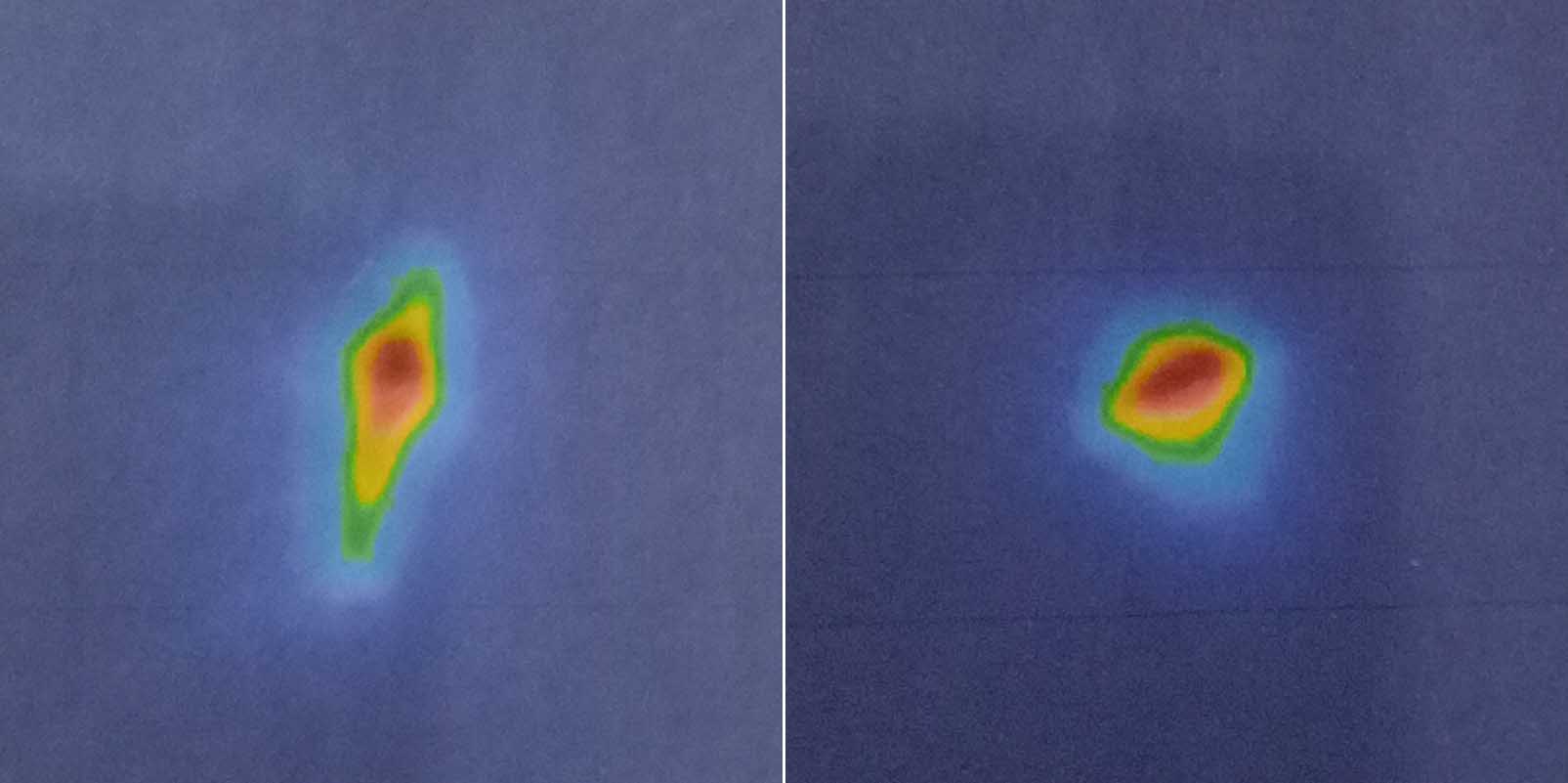** |

**Table S1:** Optical Quality Analysis System (OQAS) at one-month follow-up, indicating comparable worse vision quality in the right eye.
