# Supplementary material for: Eye damage due to cosmetic ultrasound treatment: a case report
Source: BMC Ophthalmol. 2018 Aug 29;18:214. doi: 10.1186/s12886-018-0891-2 (PMC6114535; doi:10.1186/s12886-018-0891-2)
Supplement: Supplementary file 3 — Table S2. Intraocular pressure (IOP) and uncorrected visual acuity (VA) and best-corrected visual acuities (BCVAs) of this patient. OD: right eye. OS: left eye. (DOCX 30 kb) [file 12886_2018_891_MOESM3_ESM.docx]

|  | **IOP (mm Hg)** | | **VA** | | **BCVA** | | |
| --- | --- | --- | --- | --- | --- | --- | --- |
|  | **OD** | **OS** | **OD** | **OS** | **OD** | **OS** |  |
| Pre- | **17.2** | 16.3 | **20/20** | 20/25 | **20/20** | 20/20 |  |
| Day 1 | **30.7** | 15.7 | **20/200** | 20/25 | **N/A** | N/A |  |
| Day 3 | **21.1** | 15.3 | **20/160** | 20/25 | **20/20** | 20/20 |  |
| Day 5 | **18.9** | 18.1 | **20/125** | 20/25 | **20/20** | 20/20 |  |
| Day 30 | **15.8** | 18.3 | **20/100** | 20/25 | **20/20** | 20/20 |  |
| Day 120 | **16.6** | 17.6 | **20/100** | 20/25 | **20/20** | 20/20 |  |
| Day 150 | **16.6** | 17.6 | **20/40** | 20/25 | **20/20** | 20/20 |  |

**Table S2:** Intraocular pressure (IOP) and uncorrected visual acuity (VA) and best-corrected visual acuities (BCVAs) of this patient. OD: right eye. OS: left eye.
